# Continuous Gum Dentures

**Published:** 1877-01

**Authors:** 


### CONTINUOUS GUM DENTURES.

We have often taken occasion in the pages of the REGISTER to direct the attention of its readers to continuous gum artificial dentures. It is a matter of regret that this, the most perfect method and style of constructing artificial sets of teeth, should receive so little attention, and be employed so seldom as it is. Perhaps not more than one dentist in fifty knows anything about making it, and some who have been taught the method of constructing it, ignore it altogether in practice.

Now why is this? A very common answer is, "Oh, it is too expensive." There are dentures of other materials much cheaper, so far as first cost is concerned, than continuous gum, but there is less difference in the cost than in the value.

In the difference in the expense does not, however, lie the main difficulty. This consists chiefly in the fact that so few understand anything about it, even as too its value, and much less about the mode of constructing it.

The great majority of dentists represent to their patients, that a plate of rubber with fourteen teeth set in a half circle is just as good as anything else for a denture. Some make such representations knowing them to be false; others make them under gross ignorance.

The responsibility for this condition of things rests upon the profession. A great many persons accept these miserable apologies for dentures as a last resort, knowing that there are far better things. Then again there are a great many who are ignorant of what is best, but desire to be informed and advised, and are always ready to acquiesce in the judgment and advice of those whom they regard as capable and honest.

Then again, as evidence that the people are not wholly nor even to any considerable extent, responsible in this matter, is the fact that a few men there are who use largely, and some exclusively, the best class of artificial dentures—continuous gum and gold plate work—and some of these, we know, are not situated among people different from those of communities generally.

We will use for illustration the gentleman referred to in the note below, Dr. Haskill, of Chicago, who confines himself almost exclusively to the production of the very best class of work, and his patients accept that more readily than they would the inferior and pernicious kinds of dentures in common use, and they appreciate it far more highly, and he has a far higher sense of satisfaction and gratification than he could have with any of the inferior things that are in such general use. And what is true with Dr. H. in this respect would be true of any one else who would pursue the same course.

We give with this a letter recently received by Dr. Haskill confirmatory of the views here expressed.

St. Louis, November 15th, 1876.

Dr. L. P. Haskill.

My Dear Sir:—Let me extend to you, across the three hundred miles of intervening prairie, the hand of one whose thoughts and feelings you have so exactly expressed in your late article in the Dental Register.

Would there were more of you!

Just this minute was called away from your article to see a lady and gentleman. The lady wanted a full upper and lower rubber. I talked continuous gum, but was apparently making but poor headway, when I thought of your article and said, "See what Haskill says of it." This carried the point and they are to be made of continuous gum. Yours truly,

